# ENding HIV transmission to infants by Generating Evidence to optimise prevention and care for pregnant and postpartum Adolescent Girls and young women with HIV in Tanzania (ENGAGE): study protocol for a mixed-methods research project

**DOI:** 10.1136/bmjopen-2025-115312

**Published:** 2026-06-18

**Authors:** Goodluck Willey Lyatuu, Elin C Larsson, Hellen Siril, Theodora Mbunda, Roseline F Urrio, Lameck Machumi, Daima Machang’u, Regine Unkels, Wilhellmuss I Mauka, Michael Johnson Mahande, Brenda Simba, Charles Festo, Andrew Mganga, Rashid Said Mfaume, Samwel Laizer, Honoratha Rutatinisibwa, Ayoub Kibao, Pascal Muhode, Prosper Njau, Werner Maokola, Anath Rwebembera, Michael Msangi, Mukome A Nyamhagatta, Bruno F Sunguya, Helga Naburi, Anna Mia Ekström, Anna E Kågesten

**Affiliations:** 1 Programs, Management and Development for Health, Dar es Salaam, Tanzania, United Republic of; 2 Development Studies, Muhimbili University of Health and Allied Sciences, Dar es Salaam, Tanzania, United Republic of; 3 Global Public Health, Karolinska Institutet, Stockholm, Sweden; 4 Womens and Children’s Health, Karolinska Institute, Stockholm, Sweden; 5 Center for Epidemiology and Community Medicine, Stockholm, Sweden; 6 Grants and Business Development, Management and Development for Health, Dar es Salaam, Tanzania, United Republic of; 7 Obstetric and Gynecology, Muhimbili University of Health and Allied Sciences, Dar es Salaam, Tanzania, United Republic of; 8 Strategic Information, Management and Development for Health, Dar es Salaam, Tanzania, United Republic of; 9 Epidemiology & Biostatistics, Kilimanjaro Christian Medical University College, Moshi, Tanzania, United Republic of; 10 Health, Social Welfare and Nutrition Services, Tanzania President’s Office Regional Authorities and Local Government, Dodoma, Tanzania, United Republic of; 11 Regional Administrative Secretary, Kagera, Tanzania President’s Office Regional Authorities and Local Government, Dodoma, Tanzania, United Republic of; 12 Regional Administrative Secretary, Tabora, Tanzania President’s Office Regional Authorities and Local Government, Dodoma, Tanzania, United Republic of; 13 Regional Administrative Secretary, Dar es Salaam, Tanzania President's Office Regional Authorities and Local Government, Dodoma, Tanzania, United Republic of; 14 National AIDS, STIs, and Hepatitis Control Programme, Tanzania Ministry of Health, Dodoma, Tanzania, United Republic of; 15 Preventive Services, Tanzania Ministry of Health, Dodoma, Tanzania, United Republic of; 16 Community Health, Muhimbili University of Health and Allied Sciences, Dar es Salaam, Tanzania, United Republic of; 17 Paediatrics and Child Health, Muhimbili University of Health and Allied Sciences, Dar es Salaam, Tanzania, United Republic of; 18 Infectious Diseases, Södersjukhuset Venhälsan, Stockholm, Sweden; 19 Clinical Research and Education, Karolinska Institutet, Stockholm, Sweden

**Keywords:** PMTCT, pregnancy, adolescent girls and young women, co-creation, Tanzania

## Abstract

**Abstract:**

**Introduction:**

Sub-Saharan Africa (SSA), including Tanzania, is double-burdened with high rates of teenage pregnancy and new HIV infections among adolescent girls and young women (AGYW) aged 15–24 years. Moreover, pregnant AGYW living with HIV in SSA have poorer adherence and retention on HIV treatment and elevated risks of vertical HIV transmission to their infants, as compared with older women. This paper describes the methods for the ENGAGE project, aiming to investigate and optimise healthcare for prevention of vertical HIV transmission (commonly prevention of mother-to-child transmission (PMTCT)) for AGYW living with HIV in Tanzania.

**Methods and analysis:**

ENGAGE uses a mixed-methods design to co-create and prototype an intervention package for pregnant/postpartum AGYW living with HIV through three phases in three Tanzanian regions. Phase 1 characterises the problem by investigating care engagement and outcomes in a cohort of N=10 147 AGYW receiving PMTCT services in routine healthcare. Phase 2 uses qualitative interviews to understand the social-structural drivers of care engagement from the perspective of AGYW, healthcare providers and community stakeholders and an evidence review of potential solutions. In phase 3, we will use findings from phase 1 and 2 to co-create (together with AGYW and healthcare providers) an intervention package to optimise PMTCT care for most at-risk AGYW. The co-creation will be done through an intervention development action cycle, where ideas are presented, feedback sought and refinements made iteratively via several workshops over about 6 months. The resulting co-created intervention package will be prototyped at selected facilities/communities and refined into a final version, ready for piloting for feasibility, acceptability and preliminary effect in a later phase. This protocol focuses on the co-creation phase 3 and its preceding phases 1 and 2.

**Ethics and dissemination:**

ENGAGE has received ethical approval from the Tanzania National Health Research Ethics Committee (NIMR/HQ/R.8a/Vol.IX/4637), and the Swedish Ethical Review Authority (2024-05745-01) for analysis of data in Sweden. Findings will be disseminated to AGYW, healthcare providers, community stakeholders, health officials, researchers, policy makers and the wider local and global scientific community.

**STRENGTHS AND LIMITATIONS OF THIS STUDY**

- ENGAGE employs sequential explanatory mixed methods to comprehensively understand and optimise prevention of mother-to-child transmission care engagement, retention and health outcomes for pregnant/breastfeeding adolescent girls and young women (AGYW) with HIV by first quantifying the problem, then exploring underlying reasons qualitatively, and thereafter co-create solutions with the AGYW and healthcare providers.
- A key strength is the participatory co-creation of interventions with the targeted beneficiaries (AGYW and stakeholders) ensuring their practical relevance and cultural appropriateness.
- The project benefits from analysing a large national cohort dataset; however, the findings may be influenced by variations in data quality and completeness, common in routine healthcare settings.
- Although the project aims to generate directly actionable evidence for policymakers, the focus on a single national context means the generalisability of the optimised care package to other contexts within sub-Saharan Africa may be limited.

## Introduction

 Four decades into the HIV pandemic, sub-Saharan Africa (SSA) remains the most affected region, home to 65% of the world’s almost 40 million people living with HIV and close to half of the 1.3 million new HIV infections.^1^ In SSA, two-thirds of annual new HIV infections occur among women of reproductive age, of whom adolescent girls and young women (AGYW) 15–24 years have more than three times higher risk of HIV infection than men of the same age.^2^ SSA also has the world’s highest rates of both teenage and unintended pregnancies and low rates of contraceptive use particularly among AGYW.^3^,^5^ Pregnant women with HIV who are not on anti-retroviral therapy (ART) jeopardise their survival and well-being and pose higher risk of vertical HIV transmission to their infants.^1^

Globally, among children 0–14 years old, 82% of the 120 000 new HIV infections yearly occur in SSA, with a majority (>90%) driven by vertical HIV transmission during pregnancy, childbirth or breastfeeding.^2^ Without any intervention, the risk of vertical HIV transmission can be as high as 45%.^6^ Effective use of ART among pregnant and breastfeeding women with HIV can reduce this risk to <2%.^7^ Starting all pregnant women with HIV on lifelong ART for their own HIV treatment and for prevention of vertical HIV transmission (commonly referred to as prevention of mother-to-child transmission, PMTCT) has thus been a game changer towards ending HIV infections in children.^8^ From 2010 to 2023, vertical HIV transmission declined by more than half globally due to better ART coverage,^1^ yet gaps remain, with five out of every six new HIV infections in children occurring in SSA.

Tanzania is among the twelve priority countries in the global alliance to end AIDS in children.^2^ The country has made significant progress in reducing vertical HIV transmission from 20% in 2010 to 8% in 2023, largely due to the improved ART coverage among pregnant women with HIV (98% in 2023).^2^ Nonetheless, the vertical HIV transmission rate in Tanzania is still higher than the global target of <5%.^2^

Evidence from studies in Tanzania and across SSA indicate that pregnant AGYW with HIV are lagging behind in PMTCT efforts^79^,^12^ and that their needs are not sufficiently addressed by existing standard of care.^13^ Longitudinal studies of women in PMTCT care in Tanzania found that AGYW had higher risks of poor PMTCT outcomes than older women, including twice the risk of high viral loads (>400 copies/mL) and 63% higher risk of attrition from HIV care.^7 10 12^ Similar findings have also been reported in other SSA countries.^11^ Rates of vertical HIV transmission among infants born to AGYW mothers with HIV in SSA are scarcely reported and appear to be up to three times higher than among infants of older women.^11 14^ A previous study in Tanzania observed a vertical HIV transmission rate at 18 months postpartum of 2.2% among adolescent mothers (aged <20 years) vs 1.4% among all mothers studied.^7^

Multiple risk factors affect uptake and retention in PMTCT services such as timing of ART initiation, HIV status disclosure, HIV stigma and fear of ART side effects.^13^ Social and gender norms, stigma and its socioeconomic consequences, limited resources and harmful laws disproportionately affect AGYW in the HIV epidemic, hindering their access to essential sexual and reproductive health and rights (SRHR) information and services.^14 15^

While quality evidence on AGYW is missing as previous studies mainly used age as a predictor, available data shows that the specific needs of girls and young women are not sufficiently addressed by standard PMTCT care.^11 13^ Promising strategies to improve uptake of PMTCT include youth-friendly services that emphasise confidentiality and address stigma associated with both teenage pregnancy and HIV and providing innovative models of social support, such as ‘peer mothers’ (also with HIV) accompanying women through PMTCT. However, these initiatives were typically designed *for* young women rather than in collaboration with them. Therefore, it is imperative to refine and effectively implement interventions to align with the specific needs and contextual nuances experienced by pregnant and postpartum AGYW living with HIV, including addressing unintended pregnancy, access to contraceptive services and SRHR in general.

Co-creation is a practical approach to stakeholders, such as girls and young women, healthcare providers (HCPs), community gatekeepers and researchers working together as equal partners to improve healthcare services. Co-creation has been defined as ‘a distributed and collaborative pattern of creative problem-solving that proactively mobilises public and private resources to jointly define problems and design and implement solutions’.^16^ Challenges remain in terms of how to involve most at-risk or vulnerable populations—such as AGYW living with HIV—effectively and equitably when co-creating healthcare interventions.

The ENGAGE aims to investigate and optimise PMTCT care and outcomes for pregnant and postpartum AGYW living with HIV in Tanzania. This protocol briefly describes procedures for the initial phases of the project, which aim to quantitatively assess patterns and trends of PMTCT service uptake, retention and health outcomes via analysis of routine health data (phase 1); to understand the context, drivers and potential solutions via qualitative interviews and systematic reviews (phase 2). These two preceding phases set the stage for phase 3 of ENGAGE, the main focus of this protocol, which aims to co-create and prototype a tailored package of intervention(s) to optimise PMTCT care for AGYW. A subsequent phase, which is beyond the scope of this protocol, will focus on pilot-testing the intervention package for feasibility and preliminary effect through a cluster-randomised trial.

## Methods and analysis

**Figure 1:**
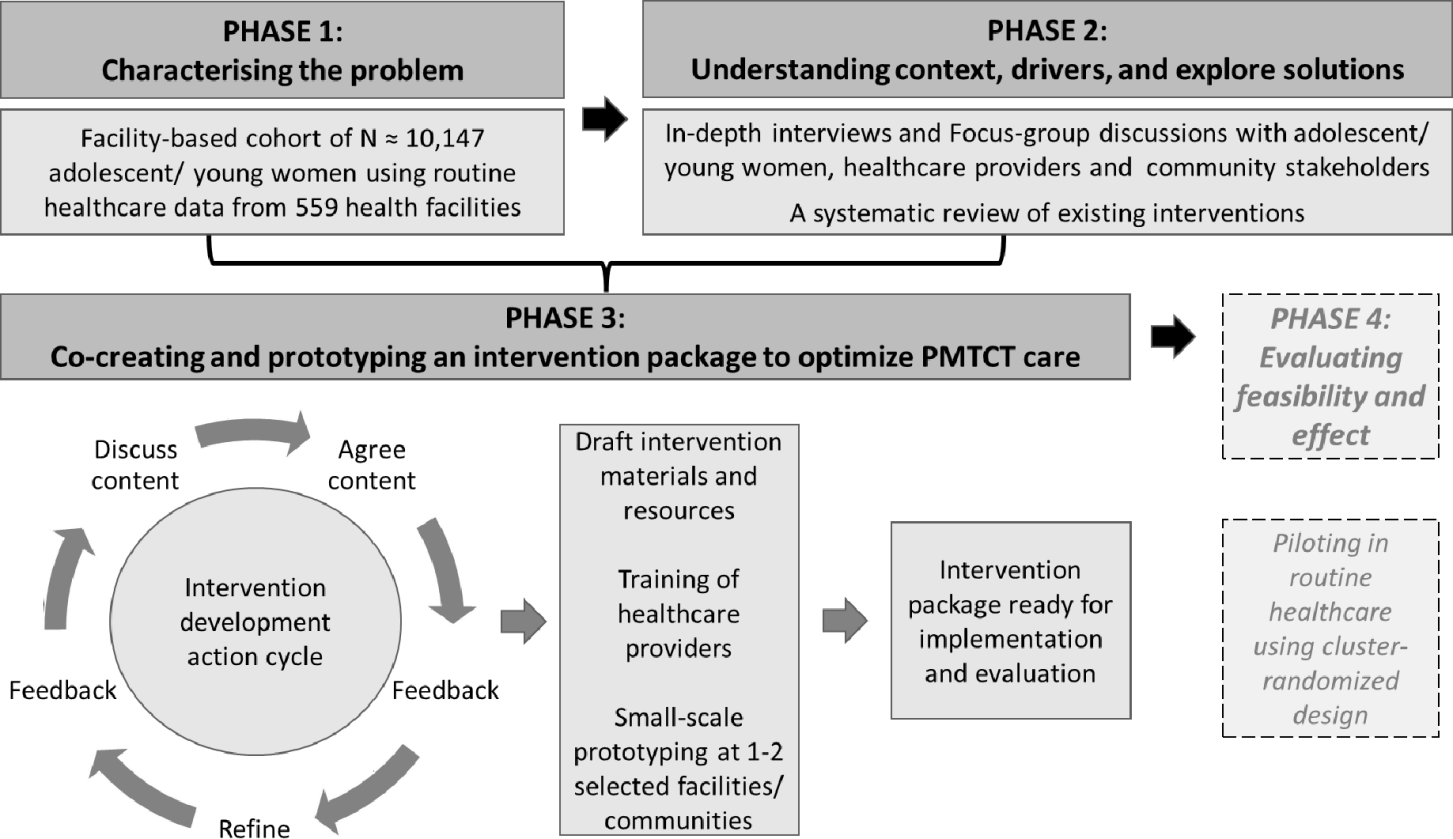
Conceptual framework, adapted from Hawkins *et al*.^16^ PMTCT, prevention of mother-to-child transmission.

ENGAGE is a mixed-methods research embedded in routine healthcare. It uses a sequential explanatory design that begins with an analysis of quantitative cohort data on AGYW enrolled in PMTCT care, followed by a stepwise qualitative inquiry to co-create, prototype and later pilot-test a package of interventions that can be easily integrated to optimise existing care approaches. Drawing on the Hawkins *et al* model for co-creating and prototyping public health interventions,^16^ the project is organised into four phases as shown in figure 1.

Phase 1 will focus on characterising the problem through a cohort study, using a large registry-based routine healthcare database on AGYW enrolled in PMTCT care in Tanzania. Phase 2 will involve qualitative consultations to understand the context and drivers of care engagement for pregnant and postpartum AGYW living with HIV and an evidence review of potential solutions available in literature. Building on findings from these activities. Phase 3 will focus on co-producing and prototyping an intervention package to optimise PMTCT care together with pregnant/postpartum AGYW living with HIV and other key stakeholders. The intervention package will later be piloted for feasibility, acceptability and effect size estimation using a randomised controlled design (in a subsequent Phase 4). This protocol focuses on phase 3 and its preceding phases 1 and 2; details on phase 4 will be published in a subsequent protocol. Preparatory activities for the study commenced in April 2023, with activities for phases 1 and 2 rolled out between 2024 and 2025, and phase 3 during November 2025–2026.

### Patient and public involvement

ENGAGE is designed to incorporate patient and public involvement during the implementation of phase 3, which entails co-designing an interventions package to optimise PMTCT care for pregnant and postpartum AGYW living with HIV. This phase involves actively working with the AGYW, young peer mothers (also living with HIV) and HCPs as designers of the interventions whereas their spouses/partners, parents and other community members are involved as the sounding board, to review and provide feedback on the co-designed interventions.

### Study setting and population

ENGAGE will be conducted in three Tanzanian regions representing both urban and rural settings: Dar es Salaam, Kagera and Tabora. Dar es Salaam is predominantly urban, the most populous and the commercial hub of Tanzania, with 5.4 million of the country’s 62 million population. In contrast, Kagera and Tabora are predominantly rural, with populations of 3.0 million in Kagera and 3.4 million in Tabora.^17^ The adult (aged ≥15 years) HIV prevalence is 4.2% in Dar es Salaam, 5.7% in Kagera and 5.6% in Tabora.^18^ Each of the three regions registers about 140 000–200 000 new pregnant women in ANC annually, of which 20%–27% are AGYW and 3%–4% are living with HIV.^19^ The study population includes pregnant/postpartum AGYW living with HIV (new, previously diagnosed and transfers) and their infants. Specific details of the target population for each phase and research activity are described below.

In Tanzania, the PMTCT care cascade starts with HIV testing of all pregnant women at ANC. Those diagnosed with HIV are initiated on a lifelong ART regimen, and their HIV viral load and CD4 count are monitored. The cascade continues after delivery with emphasis on maternal ART adherence and retention, infant ARV prophylaxis, early infant HIV diagnosis (EID), counselling on breastfeeding/infant feeding, postpartum contraceptives and psychosocial support. Women already on lifelong ART follow the same procedures.^20^ HIV care in Tanzania, including PMTCT services, is provided by the Government free of charge in public health facilities and at a subsidised cost (including free ARVs) in private facilities. The services are supported by the United States President’s Emergency Plan for AIDS Relief (PEPFAR) and the Global Fund through implementing partner organisations. At the three study regions, Management and Development for Health (MDH) is the main implementing partner supporting HIV and PMTCT services, funded by PEPFAR via the US Centers for Disease Control and Prevention. Individuals diagnosed with HIV in Tanzania are assigned a unique identification (ID) number at enrolment in HIV care and treatment clinic (CTC-ID), which is used to register, record and manage their ART care data using facility-based charts (CTC2 cards). Data from CTC2 cards are entered into the national electronic CTC2 database. On transfer between ART clinics, this CTC-ID uniquely identifies, links and tracks care and records across facilities. On delivery, infants of women with HIV are issued unique HIV exposed infant IDs, linked to their mother’s CTC-ID, and are registered in HEI cards and CTC2 database, which are used to record and track their care, including EID tests.

### Partnerships

ENGAGE is a collaborative project implemented by MDH in Tanzania in partnership with the Department of Global Public Health at Karolinska Institutet (KI), Sweden. In Tanzania, the project also engages collaborators from the Ministry of Health (MoH), the President’s Office Regional Authorities and Local Government (PORALG), Muhimbili University of Health and Allied Sciences (MUHAS) and the Regional Health Management Teams from the three study regions.

### Phase I: characterising the problem (aims 1–3)

This cohort study will use data from routine healthcare records from 559 health facilities supported by MDH, which account for over 90% of the HIV care in the three regions. Participants will comprise all pregnant/postpartum AGYW living with HIV who started PMTCT services between 1 January 2018 and 31 December 2020 and their infants born from this pregnancy/breastfeeding episode, referred to herein as the index pregnancy. Study participants will be followed for at least 2 years postpartum (until 31 December 2023) to allow enough time for final vertical HIV transmission status to be ascertained and documented (ie, 24+ months post partum). The data source will be CTC2 database, which stores data on HIV care and PMTCT services in Tanzania. Primary outcomes include: (1) time to ART attrition, defined as discontinuing ART for any reason, including death, stopping ART or loss to follow-up for >90 consecutive days from scheduled appointment; (2) detectable viral load defined as ≥50 viral copies/mL of blood after at least 3 months of ART use for those who started ART at enrolment while those already on ART detectable VL after enrolment and (3) HIV-free infant survival defined as infants who are alive and tested negative at final HIV test by 18+ months postpartum or by the last available data point. We will also evaluate repeat pregnancy, which is defined as any subsequent pregnancy occurring after the index pregnancy up to 2 years post partum. Statistical analysis will be performed using STATA software version 18, employing the Kaplan-Meier method and Cox regression models to assess time to ART attrition, HIV-free infant survival, repeat pregnancy and generalised linear models for detectable viral load. Statistical significance will be determined using a p value threshold of <0.05.

### Phase 2: qualitative consultations and evidence review (aims 4–5)

The second phase involves qualitative consultations to understand sociostructural factors influencing PMTCT care engagement for pregnant/postpartum AGYW living with HIV, and a systematic review to synthesise available evidence on potential interventions to improve the care engagement, retention and outcomes for this subpopulation (described in a separate protocol). The qualitative consultations will include in-depth interviews (IDIs) with AGYW living with HIV and their HCPs, as well as focus group discussions (FGDs) with spouses/partners of the AGYW, their parents and community stakeholders. We will use the findings of phase 1 to inform both the adaptation of interview guides/probing questions as well as the recruitment of participants for the IDIs and FGDs, which will be conducted in both urban (Dar es Salaam) and rural (Kagera) settings. Participants will be AGYW living with HIV, current in and out of care, who are currently pregnant or up to 2 years post partum at the time of the study. HCPs will be drawn from the health facilities where the AGYW came from and have been providing PMTCT services to AGYW for at least 3 years. The community stakeholders will include spouses/partners/parents/guardians of the AGYW, social workers, local community leaders, religious leaders, gender desk and teachers living within the study setting. Purposive sampling^21^ will be used to recruit participants for approximately 18 FGDs with 5–6 participants in each, and about 28–32 IDIs. The exact number of interviews will be determined based on saturation.^22^ The FGDs will be homogenous in terms of the types of participants and age. Informed consent will be sought from AGYW aged 18 years and older, mature minors (aged 15–17 years), HCPs and community stakeholders. In Tanzania, a mature minor is defined as a person aged <18 years who is a parent, pregnant, married or no longer depends on the parents (head of a household) and, therefore, does not require parental permission to participate in the activity. Data collection will be done by trained social scientists using semi-structured interview guides translated into Kiswahili. The IDIs (administered to AGYW and HCPs) and FGDs (administered to AGYW and community stakeholders) will be conducted in safe and convenient locations, ensuring confidentiality. These locations include pre-booked rooms at the PMTCT clinic, nearby school or local government offices, and sessions will be scheduled after normal working hours. We will use both deductive and inductive thematic analysis to analyse the data, which allows to draw on both theory and existing literature as well as emerging themes from the data.^23^ Two researchers will independently code the initial transcripts using a qualitative analysis software followed by discussion to develop a draft codebook. The codebook will then be refined and applied to the remaining transcripts in an iterative manner, with new codes added as needed along with regular meetings and discussions within the team.

### Phase 3: co-creation and prototyping (aim 6)

The third phase will use the results from phase 1 and 2 as a starting point and draw on transdisciplinary action research and the Hawkins *et al* framework to guide the co-creation of an intervention package to optimise PMTCT care and SRHR for AGYW.^16^ Co-creation represents a practical approach to involve stakeholders (AGYW, HCPs, community members) in improving health services.^24^ The co-creation approach aims to facilitate maximal engagement of the AGYW in designing interventions to improve their care engagement and outcomes, including shaping goals, generating and testing ideas, and influencing how these ideas impact service design and delivery.^25^ At its core, co-creation operates on the principles that everyone has the right to participate in decisions that affect their lives and that everyone possesses significant information to contribute to design processes.

**Figure 2:**
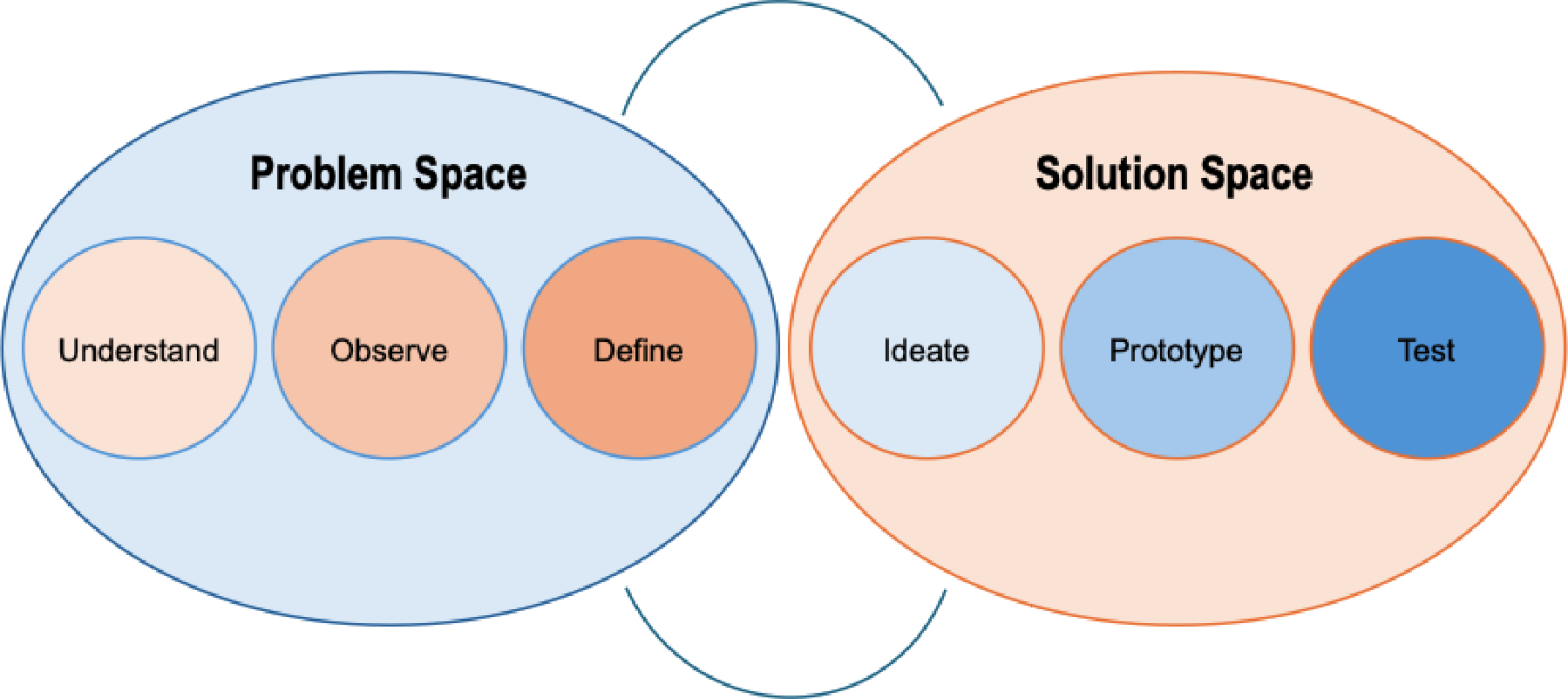
Human-centred design in co-creation.

The co-creation process will be underpinned by a human-centred design (HCD) approach, a participatory methodology that emphasised understanding the needs and experiences of users.^26^ The HCD consists of five key phases (figure 2): Understanding and Observing, where information is gathered to deeply understand user problems and experiences; Defining, where key themes are identified and problems are reframed to clarify the challenges at hand; Ideating, which involves generating actionable insights and creative ideas based on user feedback; Prototyping, where promising ideas are transformed into tangible solutions; and Testing, where developed prototypes are tested and refined based on further user input. This collaborative approach treats young people as equal partners, recognising their knowledge and abilities as valuable contributions to developing solutions,^27^ leading to empowerment, ownership, engagement and designing more culturally sensitive, contextually relevant and effective interventions.^28^

Several groups of people will contribute to the co-creation process. The core group is the designers, comprising AGYW, young peer mothers and HCPs, who will actively develop solutions during workshops. A group of sounding board members, made up of spouses/partners, parents, healthcare managers and other community members, will provide input on draft concepts and prototypes developed by the designers. The designers and sounding board members will be drawn partially from those who participated in the qualitative interviews, as well as other candidates from health facilities and communities in the target regions (health facility in-charges, local community leaders, representatives from youth organisations). Prior to their engagement, all participants will be consented on their willingness to participate in the study as described above and invited to take part in the co-creation workshops. The co-creation workshops will be moderated by a group of facilitators and note-takers who are skilled and experienced in working with young people. An intervention working group will be formed from among members of the research team and selected designers to coordinate the entire co-creation process in-between workshops.

**Figure 3:**
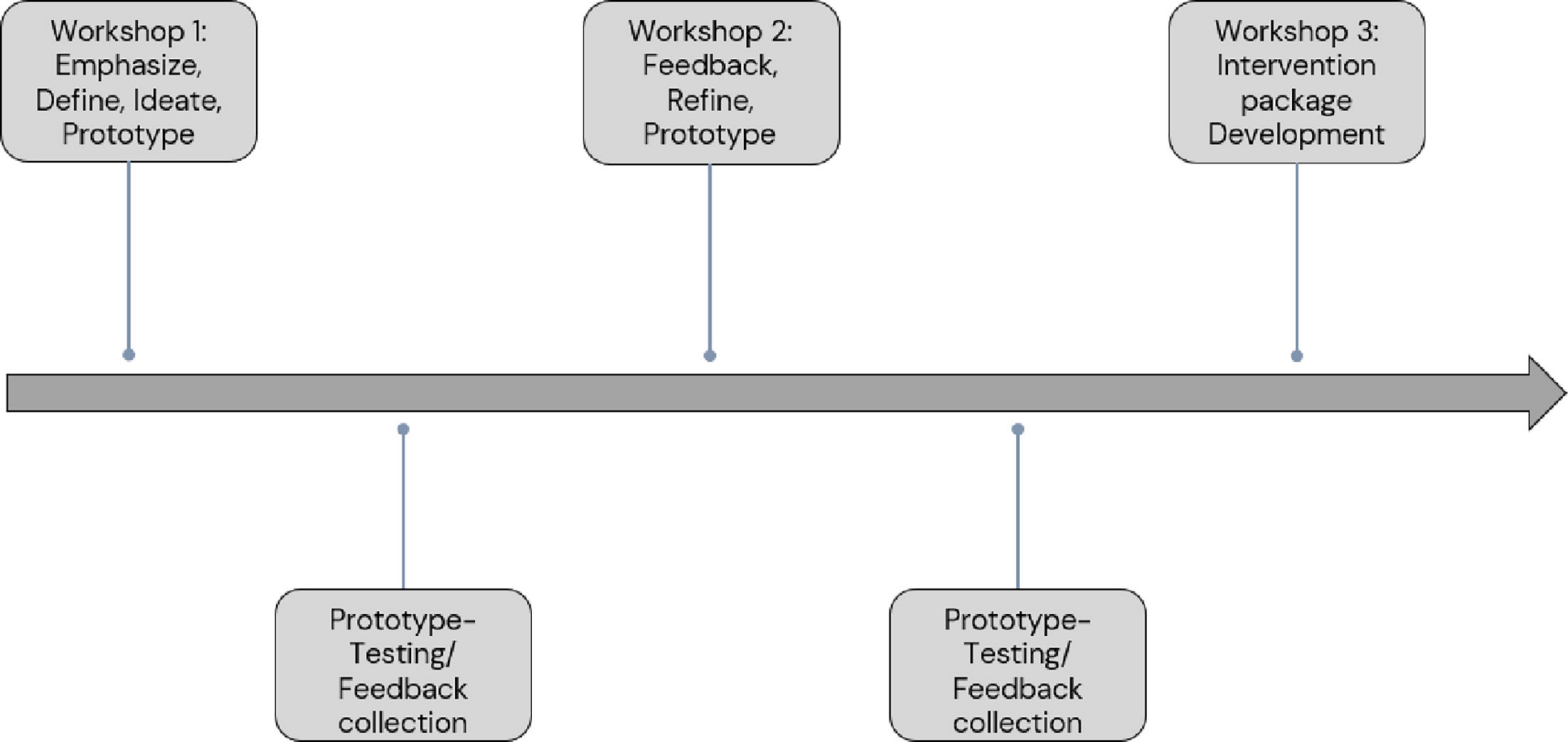
The co-creation process.

The co-creation will occur through a series of workshops (figure 3) over approximately 6 months to jointly develop the best suitable, client-centred package of interventions.^29^ This will be done via an intervention development action cycle,^16^ whereby ideas are presented by all members in the workshops, feedback is sought, and refinements are made iteratively through the preceding workshops. An intervention working group will be responsible for leading the process in between the workshops in terms of developing content and sharing it back with the broader group. The workshops will be conducted in neutral, accessible, safe venues (eg, rented training/meeting rooms) to enhance active interactions and sharpen participants’ ability to think through different creative processes.

All workshops will use inclusive and interactive methods to engage diverse participants from various sociodemographic backgrounds, such as personas, journey mapping, storyboards, role play, and brainstorming sessions. Participants will assess existing adolescent-friendly interventions related to PMTCT services, discussing their strengths, weaknesses and contextual adaptations. The workshops will be structured to promote participation, with initial sessions focusing on reviewing findings from prior studies and reframing the challenges AGYW face in accessing PMTCT services. The workshops will be conducted in homogeneous subgroups to mitigate sociocultural barriers and power dynamics. Subsequent sessions will allow participants to share experiences with current interventions, identify opportunities for improvement and brainstorm innovative, youth-centred strategies that can easily integrate into standard care. Semistructured guides will be used to facilitate in-depth discussions, while a social media group/mobile text messaging will support ongoing communication between meetings. After each workshop, the intervention working group will gather and review all discussions, ideas, and feedback to draft a package of proposed microintervention prototypes for further refinement and pretesting. These prototypes will then be tested on a small scale at one or two carefully selected intervention facilities or communities, depending on the nature of the interventions. This stage might include training of HCPs/other stakeholders to deliver the interventions (as applicable) and gaining input from AGYW and HCPs on the acceptability of the content and methods. Subsequently, the co-creation workshop participants will meet again to discuss the results and make further revisions to both the content and the delivery. This process will continue for about three or more rounds (as needed, over 6 months) until a suitable package of interventions has been formed, ready for implementation and piloting in a subsequent phase.

## Ethics and dissemination

ENGAGE has received ethical approval from the Tanzania National Health Research Ethics Committee (NIMR/HQ/R.8a/Vol.IX/4637) and the Swedish Ethical Review Authority (ref no: 2024-05745-01) for analysis of data in Sweden. The study protocol has been registered at ClinicalTrials.org (NCT06605053). Phase 1 of the project builds on routine healthcare data extracted from existing national registries. Personal identifiers such as names and physical addresses will not be collected or extracted. Data will be de-identified before analysis and stored securely in a password-protected computer to which only key investigators have access. Analyses will be reported only in aggregate form to minimise the risk of deductive disclosure.

For phases 2 and 3, written informed consent will be obtained from all participants before data collection. This includes AGYW aged 18 years and older, emancipated minors aged 15–17 years, HCPs and community stakeholders. Given the sensitivity of HIV status, pregnancy, adolescent motherhood and sexual and reproductive health, all interviews, FGDs and co-creation workshops will be conducted in private, safe and convenient locations. Participants will be informed that participation is voluntary, that they may decline to answer any question, and that they may withdraw at any time without consequences for their access to healthcare or other services. Attention will be paid to confidentiality during group-based activities, including clear ground rules for respectful participation and non-disclosure of information shared by others. The study team will use youth-friendly and participatory facilitation approaches to support meaningful involvement of AGYW while minimising potential discomfort, stigma or power imbalances. Where appropriate, co-creation activities will be conducted in homogeneous subgroups, and AGYW will be supported to contribute as active designers rather than only as study participants. Any distress or need for additional support identified during study activities will be managed through referral to appropriate health, psychosocial or community-based services available in the study regions.

Findings will be disseminated to frontline stakeholders, including AGYW, peer mothers, HCPs, facility managers and community representatives, through feedback meetings and stakeholder workshops in the participating regions. Results will also be shared with regional and national health authorities, including the MoH, PORALG and relevant HIV/PMTCT programme stakeholders, to support translation of findings into policy and practice. Dissemination to the broader scientific community will occur through peer-reviewed publications, conference presentations and engagement with local and international HIV, adolescent health, maternal health and implementation science networks.

The project is expected to generate evidence on patterns of PMTCT engagement and outcomes among pregnant and postpartum AGYW living with HIV, deepen understanding of the social and health-system factors influencing care engagement, and produce a co-created intervention package tailored to this population. The resulting package will be refined through prototyping and will inform a subsequent pilot evaluation of feasibility, acceptability and preliminary effectiveness. In this way, ENGAGE aims to contribute actionable evidence for differentiated, client-centred PMTCT and HIV care for AGYW in Tanzania and similar high-burden settings.
