# Supplementary material for: ENding HIV transmission to infants by Generating Evidence to optimise prevention and care for pregnant and postpartum Adolescent Girls and young women with HIV in Tanzania (ENGAGE): study protocol for a mixed-methods research project
Source: BMJ Open. 2026 Jun 18;16(6):e115312. doi: 10.1136/bmjopen-2025-115312 (PMC13289176; doi:10.1136/bmjopen-2025-115312)
Supplement: Supplementary file 1 [file bmjopen-2025-115312.reviewer_comments.pdf]

PEER REVIEW HISTORY

BMJ Open publishes all reviews undertaken for accepted manuscripts. Reviewers are asked to complete a checklist review form and are provided with free text boxes to elaborate on their assessment. These free text comments are reproduced below.

ARTICLE DETAILS

Title (Provisional)

ENding HIV transmission to infants by Generating Evidence to optimize prevention and care for pregnant and postpartum Adolescent Girls and young women with HIV in Tanzania (ENGAGE): Study protocol for a mixed-methods research project

Authors

Lyatuu, Goodluck Willey; Larsson, Elin C.; Siril, Hellen; Mbunda, Theodora; Urrio, Roseline F; Machumi, Lameck; Machang'u, Daima; Unkels, Regine; Mauka, Wilhellmuss; Mahande, Michael Johnson J; Simba, Brenda; Festo, Charles; Mganga, Andrew; Mfaume, Rashid Said; Laizer, Samwel; Rutatinisibwa, Honoratha; Kibao, Ayoub; Muhode, Pascal; Njau, Prosper; Maokola, Werner; Rwebembera, Anath; Msangi, Michael; Nyamhagatta, Mukome A.; Sunguya, Bruno F; Naburi, Helga; Ekström, Anna Mia; Kågesten, Anna E

VERSION 1 - REVIEW

**Reviewer**	**1**
**Name**	**Ngandu, Nobubelo**
**Affiliation**	**South African Medical Research Council, Health Systems Research Unit**
**Date**	**29-Apr-2026**
**COI**	**None**

The protocol presents a mixed methods study design for co-creating with participants, a model that would improve HIV outcomes in adolescent girls and young women living with HIV in Tanzania.

The protocol is well written, with transparency and sound methodological rigour.

Co-creating an intervention with users of healthcare is the strength of the planned study design and is likely to lead to effective outcomes, if implemented appropriately.

Phase 1 of the study overlaps with the COVID-19 pandemic period. Was there any lockdown activity during the COVID-19 pandemic in Tanzania? This would affect the said follow-up period on the database: i.e., until December 2023. Are there considerations that this period might not be well-representative of the ‘usual’ healthcare behaviour and outcomes. In the

discussion - it would be useful for the authors to explain the COVID19 situation in Tanzania at the time, and discuss the relevance or importance or potential biases related to this period.

VERSION 1 - AUTHOR RESPONSE

Reviewer 1	The protocol presents a mixed methods study design for co-creating with participants, a model that would improve HIV outcomes in adolescent girls and young women living with HIV in Tanzania.<br>The protocol is well written, with transparency and sound methodological rigour.<br>Co-creating an intervention with users of healthcare is the strength of the planned study design and is likely to lead to effective outcomes, if implemented appropriately.<br>Phase 1 of the study overlaps with the COVID-19 pandemic period. Was there any lockdown activity during the COVID-19 pandemic in Tanzania? This would affect the said follow-up period on the database: i.e., until December 2023. Are there considerations that this period might not be well-representative of the 'usual' healthcare behaviour and outcomes. In the discussion - it would be useful for the authors to explain the COVID19 situation in Tanzania at the time, and discuss the relevance or importance or potential biases related to this period.	Thank you for your positive remarks and thoughtful comments. Tanzania did not enforce a mandatory lockdown during COVID-19 pandemic, therefore the follow-up period through December 2023 remains largely representative of standard clinical practice. Nonetheless, your comment on the importance of reflecting on how the COVID19 situation may have influenced the study, when discussing study results, is well noted and will be addressed during dissemination of study findings.
